# Making evolution stick: using sticky notes to teach the mechanisms of evolutionary change

**DOI:** 10.1186/s12052-017-0074-2

**Published:** 2017-11-28

**Authors:** Teresa W. Lee, Kathleen E. Grogan, Justine S. Liepkalns

**Affiliations:** 1 Department of Cell Biology, Emory University, Atlanta, GA 30322, USA; 2 Department of Psychology, Emory University, Atlanta, GA 30322, USA; 3 Department of Biology, University of Washington, Seattle, WA 98195, USA

**Keywords:** Teaching evolution, Active learning, Natural selection, Allele frequency, Genetic drift, Gene flow, Bottleneck, Sexual selection, Evolutionary mechanisms

## Abstract

Evolution and its mechanisms of action are concepts that unite all aspects of biology, but remain some of the most difficult for students to understand. To address this challenge, we designed a hands-on activity that introduces fundamental mechanisms of evolutionary change: natural selection, genetic drift, and gene flow. In small groups, students use a population of sticky notes to reveal the consequences of each mechanism on phenotype frequency. In a followup homework assignment, students then explore how changes in phenotype frequency reflect changes in allele frequency in the population. This activity is suitable for anyone learning the basics of evolution, from high-school through the undergraduate level. We have provided detailed instructions, in-class worksheets, follow-up homework, and extensions that allow the activity to be simplified or made more complex as needed. In our own classrooms, we have observed that the concrete and collaborative nature of this activity enables students to deepen their understanding of the mechanisms through which evolution occurs. We have designed this study such that, in completing this activity, we hope to offer students the opportunity to confront potential misconceptions about evolution and gain a solid foundation for future explorations in the discipline.

## Introduction

### Background

Evolution is a unifying theory for all biological sciences (Dobzhansky 1973), and has therefore been identified as a core concept required for scientific literacy (AAAS 2011; NGSS Lead States 2013). Unfortunately, its complex and abstract nature means that evolution is one of the most commonly misunderstood aspects of biology (Gregory 2009; Taylor and Ferrari 2011). To address this difficulty, we designed a hands-on activity that uses sticky notes to visually demonstrate how evolutionary mechanisms occur.

In the United States, lack of comprehension and outright misunderstandings about evolutionary theory are magnified by the lack of public acceptance of evolution (Miller et al. 2006; Nadelson and Hardy 2015). Evolutionary concepts can be difficult to grasp because they are complex and, in some cases, seemingly counterintuitive (Coley and Tanner 2015, 2012; Richard et al. 2017) which is compounded by potential religious controversies surrounding the subject. Due to all of these factors, American students have often formed opinions and misconceptions about evolutionary theory well before entering a biology classroom which can be challenging for educators to overcome (Alters and Nelson 2002; Andrews et al. 2012; Bishop and Anderson 1990; Cunningham and Wescott 2009; Gregory 2009; Hokayem and BouJaoude 2008). Student comprehension of evolution is further confounded by the need to call upon quantitative reasoning to fully grasp the relationship between phenotype and genotype. Topics that involve math are often perceived by students as being less accessible (Betz 1978; Metz 2008), and mathematical anxiety can thwart motivation to achieve and critical thinking applications, like adapting to novel uses (Cates and Rhymer 2003; Zakaria and Nordin 2008). If left unaddressed, Gregory (2009) has demonstrated that misconceptions can endure into adulthood, where they could shape future engagement with biological research and the daunting challenges that face humanity, like antibiotic resistance (Losos et al. 2013).

In our experience, students in introductory biology classes fall into two categories: they have not learned the fundamental principles of evolution or, despite a grasp of the basics, they have an incomplete understanding of the details. For example, students may repeat the axiom that evolution is “change over time.” This simplification, although technically true, glosses over some details (e.g., evolution is allele frequency change in a population over time) that allow misconceptions to persist unexamined. Misconceptions that form early in a student’s college career can impact success in upper-division biology courses (Ingram and Nelson 2006; McKeachie et al. 2002). Although many factors affect undergraduate retention in biology, a lack of success in coursework is a key impetus for leaving the major (Chen and Soldner 2013; Cherif et al. 2014). Thus, even minor misunderstandings in introductory courses could have consequences that drive students to leave the discipline (Heddy and Nadelson 2013; Mead et al. 2015). Even more concerning, a lack of comfort with evolutionary theory may not affect all students equally-acceptance of evolution is lower among under-represented minorities, increasing the likelihood that they will avoid careers, like biology, that rely on an understanding of evolution as a foundation for success in the major (Mead et al. 2015).

Abstract concepts like evolution can be made more intuitive with the use of hands-on activities (Brewer and Zabinski 1999). Here, we present an activity that explores different mechanisms of evolutionary change using the commonly available sticky note. In small groups, students will use different colors of sticky notes to generate mixed populations. Following an in-class worksheet, students will subject their populations to different mechanisms of evolution and observe the consequences firsthand. For example, blue sticky notes may be more visible to a predator than yellow ones, and will therefore become less frequent in the population. Students will explore demonstrations of the following evolutionary mechanisms: founder effect, gene flow, genetic drift, natural selection, and bottlenecks. During this activity and its follow-up homework assignment, students will have several opportunities to directly examine any prior misconceptions about how evolution occurs. In making evolutionary theory more concrete, this activity should improve student understanding and acceptance of evolution.

We are familiar with similar activities that use different colors of manipulatives like beads or candy to represent populations consisting of different individuals. We have also used similar activities to illustrate natural selection—e.g., using plastic utensils to select for different pasta shapes. Students intuitively grasp the concept of natural selection, but struggle with the random and more abstract nature of genetic drift, making it more important to visualize the latter (Garvin-Doxas and Klymkowsky 2008; Price et al. 2016; Russo and Voloch 2012). The use of sticky notes in our activity provides several advantages. Sticky notes are larger and more visible to a lecture hall and for groups working together. They are also cost-effective to replace and easy to store and transport. An important consideration for designing this activity was that we use the same visual framework to teach many mechanisms. This feature is particularly important to emphasize that several mechanisms may be acting simultaneously on a single population. We have streamlined the counting and calculation required during group work by focusing on phenotype frequency. However, our homework assignment allows students to both revisit the mechanisms and practice calculating allele frequency.

Additionally, this activity fulfills several recommendations for best practices on teaching evolution more effectively: make extensive use of active learning, directly address student misconceptions, incorporate multimodal instruction, and introduce opportunities for communication and collaboration (AAAS 2011; Nelson 2008). The benefits of small group work and active learning have been well-documented, and are particularly effective in making theory more tangible to students (Allen and Tanner 2005; Buckberry and Silva 2012; Freeman et al. 2014; Prince 2004; Webb 1989). While the primary goal of this activity is to illustrate the effects of each mechanism, it will also demonstrate to students the metacognitive concept that using simple models can make complex subjects easier to learn.

In general, learning goals are broad statements of what an activity is intended to accomplish—these goals should be achievable, but may not be measurable. They may also describe long-term goals that require multiple activities to accomplish. Learning objectives, in contrast, describe specific and measurable learning outcomes—these are intended to be assessed, and we provide our objectives here to aid instructors in designing summative assessment questions.

### Learning goals

1. Students will know that evolution is change in allele frequency in a population.
2. Students will understand how the mechanisms of evolution change phenotype and allele frequency, and that they can act simultaneously and continuously.
3. Students will improve their collaborative group work skills.

### Learning objectives

1. Define evolution as the change in allele frequency in a population.
2. List the main mechanisms of evolutionary change explored in this activity and give an example of how each might occur.
3. Describe how each mechanism affects the phenotype and allele frequency of a population.
4. Calculate phenotype and allele frequencies in a population.

### Scientific context and rationale

Formally, evolution refers to any change in the distribution of alleles within a population over time. The concept of evolutionary change is usually introduced alongside the Hardy–Weinberg principle, a null hypothesis that describes the conditions under which evolution does not occur (such that allele frequencies remain constant between generations). Populations in Hardy–Weinberg equilibrium exhibit the following: no selection, infinite population size, no migration, random mating through sexual reproduction, and no mutation. Each of the mechanisms discussed in this activity correspond to violations of these assumptions (Table 1). These mechanisms act as drivers of evolutionary change by changing the distribution of alleles within a population (Herron and Freeman 2014; Morris et al. 2013).

Our goal for this exercise is to convey the following general concepts (Table 2): (1) Evolution occurs at the level of populations, not individuals. (2) Evolution is change in the allele frequency of a population. (3) Natural selection and genetic drift require genetic variation, which arises originally from mutation and can be augmented by subsequent gene flow. (4) Evolution commonly occurs through random chance. (5) Simple demonstrations can make abstract processes easier to understand. We designed this activity to illustrate these concepts, although they should be reinforced by lessons on evolutionary mechanisms both prior to and after this lesson. These concepts are broader than the learning objectives outlined here: as such, we hope that instructors will emphasize these concepts when teaching this lesson and draw parallels back to these concepts when teaching other lessons involving evolution.

### Development of activity and previous audiences

We first developed this activity during a mentored teaching experience as IRACDA postdoctoral fellows with the Fellowships in Research and Science Teaching program at Emory University (Brommer and Eisen 2006; Holtzclaw et al. 2005). We worked with a professor at Clark Atlanta University (CAU) to co-teach a year-long introductory biology course for majors. CAU is a private, historically black university in Atlanta, Georgia, whose enrollment is comprised of approximately 40% first-generation college students. Although this activity was originally conceived as a lecture demonstration, our class responded enthusiastically, calling out their guesses for what would happen after a particular mechanism and wincing when individuals would die. This high level of student participation prompted us to further develop the activity for small groups.

To expand its engagement and learning potential, we modified the activity for small groups and incorporated several active learning elements. We implemented this second iteration of the activity during intensive introductory biology workshops conducted at two monastic universities in India (Gaden Monastery and Sera Jey Monastery). Our students were Buddhist monks earning the equivalent of their theological doctorate. For more information about this program, please see the website of the Emory-Tibet Science Initiative (Emory Tibet Science Initiative 2016). These classes presented several interesting challenges: most of our students had never taken a modern science course, and all communication was accomplished with assistance of Tibetan translators. Like the students at CAU, however, the monks were animated participants and eagerly demonstrated their understanding of these concepts to the instructors and their classmates.

In this paper, we present this activity as we have used it, with additional ideas for tailoring this activity to each class and instructor. We have not, however, personally used all of the variations we suggest: We merely present what has worked best for us and what we believe will be effective alterations or extensions based on both scientific education literature and our own teaching experience. We encourage instructors who wish to use this activity to adjust the activity as necessary for their teaching style and availability of time and materials.

### Intended audience

This activity is flexible enough to be used in advanced high school biology classes, undergraduate introductory courses (for majors or non-majors), or upper-level classes for students majoring in evolutionary biology or ecology. Although we have used this activity in classes with 40–110 students, we believe it can be easily and effectively implemented in classes that range in size from small (≤ 10) to large (> 200). We have personally observed that this activity can successfully engage students in exploring the mechanisms of evolution across cultural and language boundaries.

## Instructional strategy

The lesson presented here is intended for a single 75-min class period, but we include extensions and variations that allow it to be tailored to the needs of individual instructors depending on time-frame, comprehension level, and class size. A general timeline is provided as a resource for the instructor (Table 3). Before this activity, students should be familiar with the following concepts: heredity, genes, alleles, haploidy vs. diploidy, genotype vs. phenotype, and mutation as a source of variation. Research demonstrates that students who first learn basic genetic concepts have an improved understanding when taught evolution (Mead et al. 2017). We summarize the lesson here, and subsequently describe each part in further detail.

At the beginning of the class period, the instructor introduces the topic of evolution with a warm-up discussion, and then guides the entire class through one example of an evolutionary mechanism: founder effect. After this demonstration, students organize into small groups in which they generate populations using four colors of sticky notes on a flat surface like the wall, floor, or a large table. Student groups are guided by a worksheet that details scenarios for each mechanism (Additional file 1). In adding and removing sticky notes of particular colors, groups see how their populations respond to each mechanism of evolution. Immediately following the activity, a synthesis discussion with the whole class allows the instructor to address any areas of confusion and ensure that all students have a clear understanding of each mechanism. The provided homework worksheet (Additional file 2) allows an opportunity for students to integrate an understanding of allele frequencies with the phenotypic changes they observed during the group work.

### Before class

Instructors should familiarize themselves with the teaching strategy and learning objectives, modifying these for their class level and size. If needed, they can modify the in-class worksheet (Additional file 1) to cover the desired mechanisms, and print out one copy for each student. They should purchase at least four colors of sticky notes, with enough notes for each group to have 30 notes of each color. A standard pad of sticky notes has 100 notes.

Before the activity, students should already understand genetic concepts like alleles, genotype, and phenotype. As homework before the activity, students should read the section of their text introducing evolution and the mechanisms by which it occurs. If the class is not using a textbook, the instructor can have students watch a short instructional video online (a useful video is BioFlix 2009). They should then write short responses to the warm-up questions in Table 4, and come to class prepared to discuss these questions. The goal of these warm-up questions is to motivate the students to begin thinking about the concept of evolution, which can be achieved by other assignments (e.g., readings, multiple choice questions, etc.) according to the instructor’s preference.

### During class

#### Warm-up discussion

Before beginning the group work, instructors should solicit responses to the warm-up questions. Depending on class attitudes and composition, this can be done in a whole-class discussion, in which students can seek input from their peers and the instructor. Alternatively, responses can be discussed in smaller groups, which will allow students to have more opportunity to articulate their thinking while discussing with their peers. If instructors feel that students may be reluctant to share their answers, they can ask students to turn to their neighbor and discuss their answers for a few minutes before initiating a larger discussion (Think-Pair-Share). This warm-up will allow students to demonstrate their prior knowledge, practice self-explanation, and may expose some misconceptions. The instructor can identify these and address them either at this time or during the post-activity synthesis discussion. If this warm-up is conducted in small groups, we encourage instructors to circulate among groups to get a sense of student understanding. We have highlighted some common misconceptions in Table 2 (AAAS Project 2061 2017; Bishop and Anderson 1990; Coley and Tanner 2012; Nehm et al. 2010; Nehm and Reilly 2007; Petrosino et al. 2015; UCMP and NCSE 2012; Yates and Marek 2014). Those misconceptions that are specifically addressed in this activity are marked by asterisks. Instructors may wish to refer to Padian (2013) for a more general discussion about the language and interpretation of evolution. Although students may feel that they have adequate answers to these questions, the activity should prompt them to provide more detail when revisiting the questions. By discussing these warm-up questions, students will begin thinking about the key concepts of this activity as they begin the group work.

#### Introduce activity

Each student should have a copy of the accompanying inclass worksheet (Additional file 1). If students have not previously worked in groups, the instructor may find it helpful to begin with an explanation of why this activity will be valuable for them, with a focus on the benefits of group work. Instructors can refer to Felder (2007) for ideas of how to improve student buy-in for active learning methods. This explanation will not be necessary for those who frequently employ active learning in their courses, in which case instructors can share the learning objectives and proceed with the activity.

**Fig. 1:**
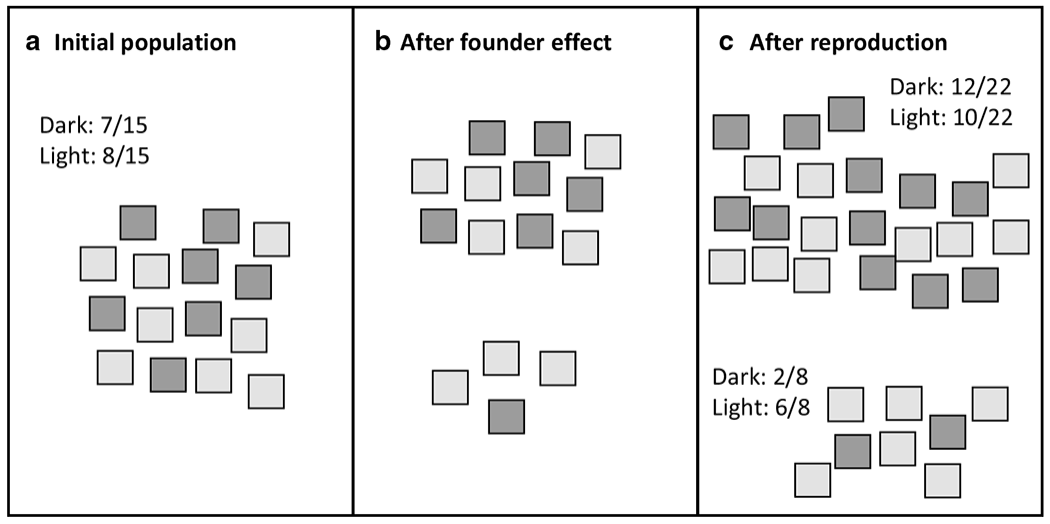
Detailed diagram of instructor demonstration, including the appearance of the population after a round of reproduction. **a** Initial population with corresponding phenotypic frequencies. **b** After founder effect. **c** After reproduction with corresponding phenotypic frequency

Instructors begin by introducing their newly discovered species: the Sticky Note. This species is exceptionally long-lived, so one note can reproduce for many generations. Each note represents one haploid individual in a population. Each individual has one important physical trait—color—determined by the particular allele possessed by that note. We have provided Fig. 1 as a reference of this demonstration. At the front of the room, the instructor generates a population of ~ 15 notes on the board with two colors in a roughly 1:1 ratio (Fig. 1).

During this time, the instructor can review the definitions of ‘gene’ and ‘allele,’ using the sticky notes as examples. Once the population is established, the instructor walks the class through calculating the phenotypic frequencies of the alleles in this population (Fig. 1a). Then, the instructor (or a student volunteer) moves a few sticky notes over to a nearby island (Fig. 1b), demonstrating the founder effect by creating a separate, smaller population. At this time, they may want to make a mark on each note—this helps distinguish the notes who were members of the original population from those notes’ progeny. We found that, without distinguishing marks, it can be difficult to keep track of which notes had already reproduced. Each sticky note then reproduces clonally (Fig. 1c). The instructor guides the class through calculating the new phenotypic frequencies and comparing them to those of the initial population.

#### Complete activity in small groups

**Fig. 2:**
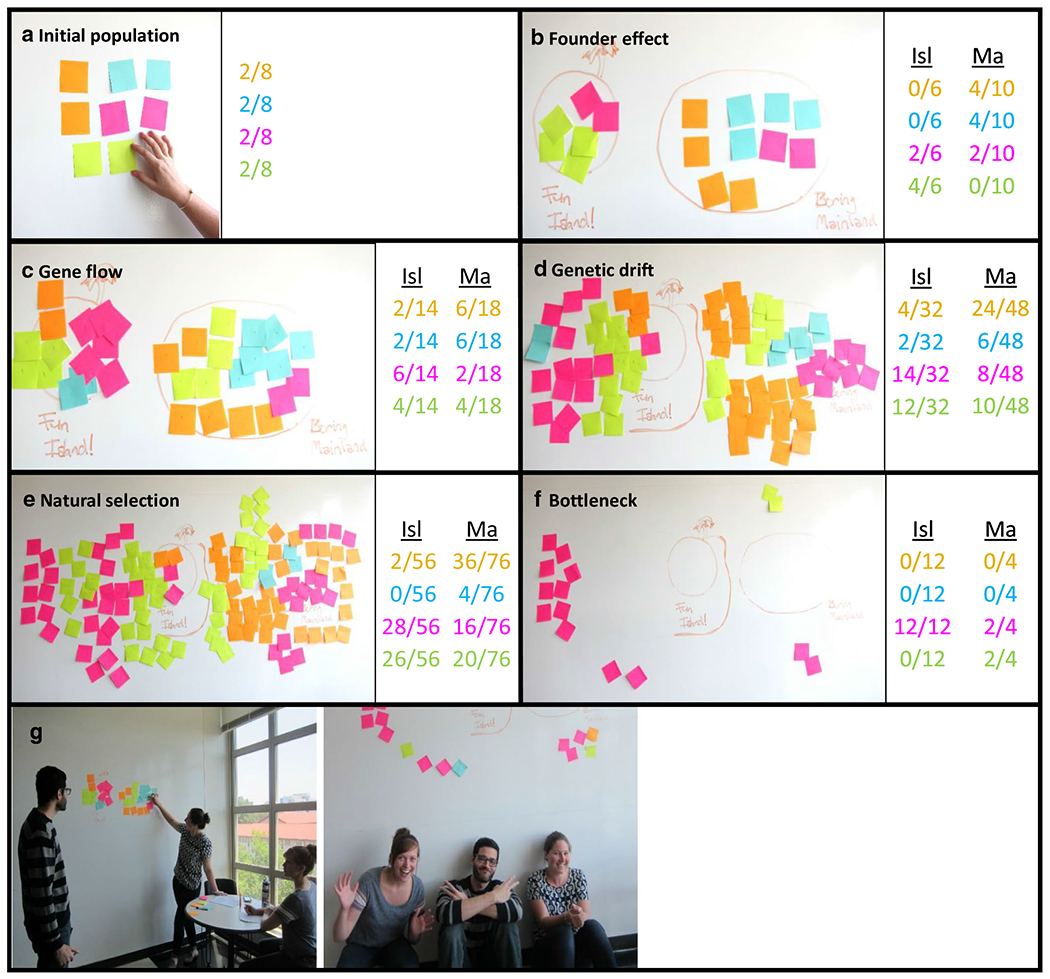
Example of mechanisms during small group activity. This figure is intended to correspond with the worksheet in Additional file 1, using four colors of sticky note: orange, blue, pink, and green. **a** Initial population. **b** After founder effect from Boring Mainland (Ma) to Fun Island (Isl) + reproduction. **c** After gene flow + reproduction. **d** After genetic drift + reproduction. **e** After natural selection from predation of orange and blue notes + reproduction. **f** After bottleneck from alien bombs (impact sites determined by tossing balls of paper at the board) + reproduction. **g** Photos of activity implementation

Students break into their small groups to work through the activity. In our classes, we have found that groups ideally will consist of three or four students, although the group size should depend, in part, on the size of the class as well as the materials and wall space available. We have provided Fig. 2 as a reference of what sticky note populations might look like after groups move through each of the steps in the worksheet (summarized in Table 5). On their own, groups establish an initial population of eight individuals using four colors of sticky notes, two of each color (Fig. 2a). If possible, students could use a whiteboard, chalkboard, or easel paper as their surface, which allows them to annotate around their sticky note population. They first reconstruct the founder effect (Fig. 2b). Throughout the rest of the exercise, the instructor can decide how to proceed through the mechanisms—we illustrate one method here, which follows our in-class worksheet (Additional file 1). Following the establishment of two populations via the founder effect, student groups then explore gene flow by moving a few sticky notes from the mainland to the island or vice versa (Fig. 2c). To show genetic drift, one student closes their eyes and randomly removes the same number of sticky notes from each population (Fig. 2d). To examine natural selection, another student in each group picks two colors that they especially like, and remove notes of these colors from both populations (Fig. 2e). Finally, the group decides on an event of mass destruction that eliminates a large portion of each population (hurricane, earthquake, plague), and a last group member demonstrates the bottleneck effect by removing many notes (Fig. 2f). Depending on the disaster, sticky notes can be removed in several ways: at random, according to geographic location (e.g., closer to the ocean vs. farther away), or according to color (e.g., susceptibility to disease). As the groups move through the exercise, each student will complete their own worksheet of phenotypic frequencies calculated for both mainland and island populations.

If desired, instructors can choose to have groups take photos of their populations (initial population and after each event) to submit along with the in-class worksheets. These photos will allow instructors to determine whether groups are correctly calculating phenotypic ratios. We also suggest that, after the activity, instructors ask students to walk around the classroom to see how the populations of other student groups were similarly or differently affected by sequence of events. Alternatively, the instructor could keep time for the whole class, such that every group is working on the same mechanism at the same time. If this is the case, students can walk around between each mechanism to see how different groups’ populations responded to each mechanism. In other words, students can see how the same starting population leads to different evolutionary outcomes based on the way the different mechanisms were enacted: For example, moving only yellow sticky notes to the island during the founder effect demonstration may result in very different phenotypic frequencies on the island and mainland than a group that moved a random assortment of sticky notes to the island.

#### Class synthesis

After the completion of group work, instructors should bring the whole class together for a synthesis discussion. We have generated questions that allow students to revisit the warm-up questions and discuss the ramifications of these evolutionary mechanisms (Table 3). Discussion should emphasize that the survival of an individual only contributes to evolution if that individual reproduces, therefore passing its alleles onto the next generation. Additionally, instructors can point out that in nature, these mechanisms can occur simultaneously. They should highlight the effect that population size had on each population’s phenotypic and allelic diversity, and discuss the implications of evolution by selection compared to evolution by genetic drift. This discussion also provides a good opportunity for students to bridge the concrete examples of their sticky note populations with the abstract definitions they wrote on their in-class worksheet (Additional file 1)—instructors could have students define each mechanism within the context of their simulated populations. While implementing this activity, we noticed a lingering misconception that should also be addressed in this synthesis: Individuals are able to evolve, or ‘develop an adaptation,’ if they are faced with a specific challenge. Because of the collaborative nature of this activity, we were able to identify instances when students slipped into language that indicated an individual’s agency, such as “It needed to…,” “In order to…,” or “To become….”

### After class

To further extend and cement the concepts presented in this activity, we have provided a homework assignment that introduces allele frequency calculations (Additional file 2). This assignment will allow students to apply what they have learned in class, while asking them to integrate their understanding of how genotype relates to phenotype. In this assignment, students are now observing a different species of *diploid* sticky notes, whose color is determined by two alleles, “A” and “a.” These notes have three phenotypes: purple, gray, and white (which are still distinguishable even if worksheets are printed in gray-scale). To avoid confusing the “species” of sticky note used for the class activity with the species used for the homework, we have used a triangle shape of sticky note for this worksheet. Students must now calculate allelic frequencies for images provided in the assignment. After completing the homework, they should be prepared for lessons on population genetics and the Hardy–Weinberg equilibrium equation. As students may struggle with the transition of calculating allelic frequencies for diploid organisms, students can be asked to complete this homework in their groups outside of class. Alternatively, the instructor may wish to review at least a few questions on the homework in the next class, or post the answer key for the students to review outside of class. If Supplemental Assignment #2 is used as an in-class activity, students can also use skittles (or other candy) to move around on their desks (to represent the moving triangles). We have found students engaged by having to physically move the colored candy.

### Implementation tips for in-class activity

If possible, give each group whiteboard or chalkboard space. This area will allow groups to draw around their populations and make notes. If this option is not feasible, consider obtaining large easel pads, and having each group use two or three large sheets as backdrop for their sticky note populations. We have found that normal sticky notes work well for this activity, but depending on the surface to be used, instructors could purchase sticky notes with a stronger adhesive to help with sticking. To impose some structure on which student will be responsible for moving sticky notes during each mechanism, instructors can ask students to take turns in a certain order (e.g., shortest to longest hair length, order of birthdate, height).

### Variations on in-class activity

1. Think-Pair-Share method: We strongly encourage instructors to implement this activity in small groups. However, if time or space are limited, this activity can be used without placing students into small groups, but rather as a series of think-pair-shares with the class as a whole. The instructor prepares the activity on the board (as previously described). The instructor then asks students to consider how the sticky notes would either move, change, or be removed during an evolutionary process by drawing on their notebooks. The students then share their answers with their neighbors. Lastly, the instructor asks someone to share their answer with the class by coming to the board and moving the sticky notes directly. This Think-Pair-Share is carried out for each stage of the activity to demonstrate the different evolutionary processes. The instructor or student volunteers demonstrate each mechanism at the board during the “sharing” part of the think-pair-share. The students do the calculations of the phenotypic frequencies on their individual worksheets.
  This modification simplifies the logistics and minimizes the time required for student-driven group work. If this is the case, consider buying mediumsized sticky notes for better visibility, which may be especially important for large lecture halls. We originally implemented this activity in this manner and students were enthusiastic observers—calling out answers, asking questions, and displaying their preferences towards particular sticky note phenotypes. However, based on our experience and that of others, this activity would be most effective when performed by small groups – students are more engaged if they are physically involved in the movement of the sticky notes (Price 2011; Springer et al. 1999). Small group work also allows students to have fun with the activity, deciding which natural disaster will occur and arguing over which colors should be favored by natural selection (although in our whole-class discussions, there was still vigorous debate about the fates of particular sticky notes).
2. Jigsaw method: If space and time are limited, but the instructor would like to keep the small group aspect of this activity, we suggest using a modified jigsaw method (Aronson et al. 1978; Social Psychology Network 2000). Instructors should first demonstrate one mechanism (e.g., founder effect) to the whole class as in the original activity design. As a compromise between student agency and activity complexity, students could be separated into ‘expert’ groups tasked with enacting a single mechanism: Group A is assigned gene flow, Group B is assigned drift, and so on. Groups would then be rearranged into ‘jigsaw’ groups, consisting of one member representing each mechanism: Group 1 has a member from A, a member from B, and so on. In their jigsaw groups, each expert will teach their group members about their particular mechanism. If instructors choose this option, we recommend having groups only explore four mechanisms: gene flow, genetic drift, natural selection, and bottlenecks. To simplify this extension further, instructors could simply have each expert group explain their scenario and mechanism to the entire class.
3. Subset of mechanisms: To decrease complexity of the activity using any of these variations, the instructor can also choose to focus on a subset of the evolutionary mechanisms described here.

### Activity extensions

1. To reinforce the mechanisms discussed in this activity, we suggest creating a review activity in which instructors show the class diagrams of populations before and after a mechanism has occurred in the population. Students will explain which mechanism is shown and why they came to that conclusion. This would also be a useful alternative to the homework worksheet (Additional file 2) if instructors choose not to cover allele frequency calculations.
2. To make this activity more challenging, the instructor could modify the in-class worksheet to include sections that demonstrate the simultaneous action of multiple mechanisms. The small groups would then cover each mechanism acting alone and some acting in combination.
3. As a more in-depth follow-up to this activity, students can complete a written assignment identifying actual examples of each mechanism in nature. Their examples should include the population, the mechanism(s), and consequences for each population. This assignment would allow students to bridge the concepts covered in this activity with real-world occurrences of evolution.
4. As preparation for a quiz or exam, consider a review where the class revisits a population of diploid sticky notes. In this review, multiple mechanisms can act individually or simultaneously upon the population. Student volunteers are each assigned a different role in the sticky notes’ ecosystem:
  - An earthquake (bottleneck).
  - A predator that only harvests from the edges (natural selection).
  - A predator that only eats notes of a certain color (natural selection).
  - A sandbar that allows some sticky notes to leave or join the population (founder effect or gene flow).
  - A mutation that can affect reproduction, fitness, or sticky note color.
  Other students can vote, roll dice, or draw numbers to choose multiple mechanisms that will act on a population. The class should write down their prediction of what will happen, and this prediction will either be confirmed or corrected when the chosen volunteers act upon the population.
5. To evaluate student preparedness before the lesson or to evaluate learning after the lesson, instructors may wish to administer the Conceptual Assessment of Natural Selection (Kalinowski et al. 2016) or the Genetic Drift Inventory (Price et al. 2014). These inventories might also provide useful guidance for designing exam questions.

## Conclusion

In our classes, we found that students often struggled with understanding how evolutionary mechanisms affect individuals and populations. Some mechanisms are intuitive: for example, the founding of a new colony or a natural disaster bottleneck. Other mechanisms, like genetic drift, are considerably more difficult to grasp. Students also had trouble connecting these mechanisms to their effects on allele frequency within a population, and sometimes failed to make the leap that connects genotype to phenotype in the context of population genetics. The goal of this activity is to address these challenges by providing a visual representation of how populations respond to each evolutionary mechanism. We have included warmup questions, synthesis questions, homework, and review options that all work together to reinforce our learning objectives.

Our activity relies on a student-centered instructional method as opposed to the traditional instructor-centered lecture style. Students themselves become each mechanism, acting out its effects on their population of sticky notes. Their physical participation in this activity helps to diffuse some of the tension that commonly accompanies learning about evolution (Price 2011). In developing this activity, we have worked with two very different types of students: first-year undergraduates at a historically black university and adult Buddhist monks. Despite their cultural and educational differences, both groups relished the opportunity to move the sticky notes themselves. Students seemed to enjoy the agency of “being evolution” and making decisions that would affect their populations. The concrete nature of this activity, along with the accompanying discussions and homework, employs multiple learning modalities—auditory, tactile, and visual—to reach a wider range of students and learning styles. In our course evaluations, most students mentioned that either “group activities” or “hands-on activities” helped them acquire the knowledge covered during the course. We found that a primary advantage of this activity is that it makes student conceptualizations visible, which allows peers or instructors to respond immediately to misconceptions. This advantage is further supported by the use of small groups, which fosters a supportive learning environment in the classroom. Working in groups allows confident students to make their knowledge explicit, while struggling students can get help from their peers.

There is overwhelming evidence that hands-on activities are more effective than lectures, in part because students become active participants in their own learning (Alters and Nelson 2002; Hake 1998; Nelson 2008; Smith et al. 2005). Recent standards have recommended that biology classrooms use more interactive and cooperative styles of teaching, which offer more opportunities for students to practice critical thinking (AAAS 2011; Nelson 2008). These standards have also identified evolution as a core concept to address in all biology classes. This activity is well-aligned with these broader goals for biology education. The assessments we have designed help students construct their own models of population change, and apply them both qualitatively (in-class worksheet and review activities) and quantitatively (homework on calculating allele frequencies). After this lesson, students should be prepared for further explorations in understanding how evolutionary change occurs in our world.

## Figures and Tables

**Table 1:** A summary of the main mechanisms of evolutionary change. Mechanisms are listed in the order presented in the activity (Futuyma 1998; Morris et al. 2013)

Mechanism	Description
Founder effect	An event that occurs when a fraction of the members of a population leave the main population to form a secondary population. Just as in bottlenecks, allele frequency in the colony may differ from the main population, but this is due to colonization and not catastrophe. Can be a specialized instance of genetic drift
Gene flow	The transfer of alleles between populations, usually through migration of an individual or its gametes. Gene flow is one of the main mechanisms, in addition to mutation, and recombination, that can introduce new genetic variants to a population
Genetic drift	Random fluctuations in allele frequency, due to chance or random sampling; its effects are more obvious in smaller populations.
Natural selection	Occurs when individuals with a heritable trait have higher fitness (via increased survival and/or offspring number) than individuals without the trait. Individuals with the trait then pass their alleles to their offspring. This is a non-random process. Over generational time, selection can create populations that have adapted to succeed in specific environments
Population bottleneck	An event in which a population’s size is severely reduced, e.g., by a natural disaster like volcanic eruption or disease like the Black Plague. Allele frequencies in the surviving population may differ from those in the original population, and some alleles might be missing altogether
Sexual selection (only covered in homework)	A specific instance of natural selection. Evolution by sexual selection occurs when individuals with a heritable trait are more successful at getting mates and producing offspring than individuals without the trait
Mutation (not addressed in activity)	Refers to changes in an individual’s genome. Provides the ultimate and original source of genetic variation for a species or population. Not all mutations have consequences for an individual (neutral mutations), but those that do can be harmful or beneficial. Must occur in the gametes of an organism to be a mechanism for evolution (i.e., the change must be heritable)

**Table 2:** Central concepts and common misconceptions. A. Central concepts of the activity B. Common misconceptions about these topics that instructors should be sure to address (asterisks mark those specifically addressed by this activity). References are included where applicable

A. Central concepts	1. Evolution does not occur in individuals, only in populations	
	2. Evolution is change in a population’s allele frequency	
	3. Natural selection and genetic drift require genetic variation, which arises from mutation and gene flow	
	4. Evolution can occur through random chance	
	5. Simple demonstrations can make abstract processes easier to understand	
B. Common misconceptions	1. All traits of organisms are adaptations	Anderson et al. (2002)
	2. All members of a population develop new traits at the same time	
	3. “Fitness” refers to the strength, size, or speed of an individual	Padian (2013)
	4.* Evolutionary mechanisms serve a purpose or strive for perfection. Natural selection involves individuals trying to adapt. Natural selection gives organisms what they need	Bishop and Anderson (1990), Anderson et al. (2002) and Nehm et al. (2010)
	5.* Individuals can evolve. “Adaptation” means adjustment within an individual’s lifetime	Bishop and Anderson (1990)
	6. All mutations are harmful	Anderson et al. (2002)
	7.* Natural selection and evolution are the same thing	Bishop and Anderson (1990) and Nehm and Reilly (2007)
	8.* Genetic drift only occurs in small populations	Price et al. (2014)
	9.* Evolution is slow and gradual	Price et al. (2014)

**Table 3:** Activity timeline for instructor

Activity	Description	Approximate time (min)
*Before class*		
Instructor prep	Review concepts and modify activity as needed. Prepare handouts. Procure sticky notes (four colors, with ~ 30 notes of each color per group)	60–120
Student prep	Read assigned section of text. Write short answers to pre-activity discussion questions (Table 2B)	15–30
*During class*		
Warm-up discussion	Whole-class discussion on pre-class questions (Table 2B)	10
Introduce activity	Instructor describes sticky note species and demonstrates mechanism of founder effect	5
Complete activity in small groups	Spend about 6 min enacting each mechanism; each student also completes individual handout (Additional file 1)	45
Wrap-up and group rearrangement	During this time, students can walk around to examine other groups’ populations	5
Synthesis discussion	Whole-class discussion to address misconceptions (Table 2C) and synthesis questions (Table 2D)	10
*After class*		
Homework	Complete worksheet that reviews material and introduces allele frequency calculations (Additional file 2)	20–45
Review	See extensions: guess the mechanism, apply multiple mechanisms	15–30
*Variations*		
Think/pair/share	A time-saving alternative to small-group work. The entire class focuses on one population. Student pairs use the think/pair/share method to follow along on their individual handout (Additional file 1), which student volunteers coming to the front of the room to demonstrate each mechanism	20–30
Jigsaw method	Activity will cover four mechanism and be divided into two parts: students should spend about 10 min in their expert groups and 20 min in their novice groups (spending about 5 min per mechanism); 10 min are built-in for rearranging groups	40

**Table 4:** Questions to ask before and after the activity. A. warm-up questions to ask of the whole class or in smaller groups, and B. synthesis discussion questions to ask of the whole class

A. Warm-up questions	1. Define evolution in your own words
	2. Is evolution happening today?
	3. What causes evolution to happen in nature?
B. Synthesis questions	1. What characteristics could cause one color of sticky note to survive or reproduce better than another color?
	2. What does fitness mean in the context of evolution?
	3. How is evolution related to genes?
	4. Does evolution occur if an individual migrates but dies before reproducing?
	5. What are the consequences of allelic fixation?
	6. Has this activity changed your views on evolution? Why? a. Does evolution occur today? b. What causes evolution to occur in nature? c. What is the most important driver of evolution?
	7. How does natural selection differ from the other mechanisms of evolution?
	8. Name a contemporary example of an evolutionary process
	9. Are humans currently evolving?

**Table 5:** Summary of instructions on the in-class worksheet (Additional file 1) to generate populations seen in Fig. 2

Evolutionary Mechanism	Description	Student Instructions
1. Discover population		Establish a population of sticky notes with 2 of each color
2. Founder effect	Some curious sticky notes explore a nearby island and get stuck there	Move a few notes of any color to the island (no more than 3) Clonal reproduction: For each Note on the island and the mainland, add another of the same color to the same population
3. Gene flow	Periodically, some sticky notes can swim between the mainland and the island	One member of your group will choose up to 5 intrepid sticky notes to move from one population to the other—some can move to the mainland, while others can move to the island Clonal reproduction
4. Genetic drift	You, the researcher, leave the population, and are gone for several generations	One member of your group will close their eyes and remove 2 Notes from the mainland population and 2 from the island population Clonal reproduction Again, the group member closes their eyes and removes 8 Notes from each population Clonal reproduction
5. Natural selection	Sticky notes have a dreaded flying predator that prefers two of their colors	A group member flies between the island and the mainland, removing 10 notes of these two colors (decide how many to eat from each population) Clonal reproduction
6. Bottleneck	An event of mass destruction (group choice!) decimates the population	Remove all but 10 notes (decide how to split survivors between populations) Clonal reproduction

## Footnotes

Additional file 1. In-class worksheet and key.

Additional file 2. After class homework worksheet and key.
